# Investigation of Coronal Leakage of Root Fillings after Smear Layer Removal with EDTA or Er,Cr:YSGG Laser through Capillary Flow Porometry

**DOI:** 10.1155/2014/593160

**Published:** 2014-02-19

**Authors:** Tom Edgard Maria Vergauwen, Rafaël Michiels, Dries Torbeyns, Maarten Meire, Mieke De Bruyne, Roeland Jozef Gentil De Moor

**Affiliations:** Department of Restorative Dentistry and Endodontology, Ghent Dental Laser Centre, Ghent Dental Photonics Research Clustre, Dental School, Ghent University, De Pintelaan 195/P8, 9000 Gent, Belgium

## Abstract

No studies have been performed evaluating the marginal seal of root fillings after direct exposure of root canal (RC) walls to Er,Cr:YSGG laser irradiation. Therefore, 75 root filled teeth (5 × 15–cold lateral condensation) were analyzed for through-and-through leakage (TTL) using capillary flow porometry (CFP). The cleaning protocol determined the experimental groups: (1) irrigation with NaOCl 2.5% and EDTA 17% or standard protocol (SP), (2) SP + Er,Cr:YSGG lasing (dried RC), (3) NaOCl 2.5% + Er,Cr:YSGG lasing (dried RC), (4) SP + Er,Cr:YSGG lasing (wet RC), and (5) NaOCl 2.5% + Er,Cr:YSGG lasing (wet RC). Groups 6 to 10 consisted of the same filled teeth with resected apices. Resection was performed after the first CFP measurement. CFP was used to assess minimum, mean flow, and maximum pore diameters after 48 h. Statistics were performed using nonparametric tests (*P* > 0.05). Additional three roots per group were submitted to SEM of the RC walls. TTL was observed in all groups without statistically significant differences between the different groups for minimum, mean, and maximum pore diameter (*P* > 0.05). In this study, the use of EDTA and/or Er,Cr:YSGG laser did not reduce through-and-through leakage in nonresected and resected roots.

## 1. Introduction

Erbium lasers are mostly used because of their photoablative action similar to that of cavity preparation (thermomechanical tissue interaction). Because water has the strongest absorption peak for electromagnetic radiation at around 2,900 nm, erbium lasers (Er:YAG—2,940 nm; Er,Cr:YSGG—2,796 nm) emitting at around this wavelength are a suitable instrument for ablation of dentin [1–3].

Experimental studies when using the Er:YAG laser fiber for direct exposure to root canal walls have demonstrated that this type of laser is more effective in removing the smear layer than other laser types and endodontic irrigants [4, 5]. The dentinal walls mostly show open tubules [6–8] and are free of debris or a smear layer [6, 7].

As the laser fiber is used in a circular motion whilst withdrawing the optical fiber (this withdrawal might otherwise have been slower or even have halted in certain areas), in some of the areas irradiated, not all of the tubules are completely open [9, 10]. Differences in power settings do not appear to result in significant differences in efficacy for removing the smear layer [7, 11, 12]. Although Er:YAG laser irradiation is reported to be effective for removing debris and the smear layer [6, 7], a better apical seal is not necessarily achieved [13, 14].

Depending on the type of sealer used, adhesion to the root canal wall was increased or remained unchanged [15]. When comparing Er:YAG laser and EDTAC solutions, both means of cleaning the root canal wall increased the ability of root canal sealers containing calcium hydroxide to adhere to human dentin [16].

In 2002, the US Food and Drug Administration (FDA) approved the Er,Cr:YSGG laser for use in conventional and endodontic therapy. Contradictory results for root canal wall cleanliness are found as follows: more remaining debris than with a conventional technique (root canal preparation and irrigation, no lasers used) [17]; no significant differences [18]; heterogeneous debris removal with partial and total removal of dentinal debris, as well as a few sites showing thermal injury including carbonization and partial melting [19, 20]; and a better cleaning ability than NaOCl [19, 21]. Differences in power outputs, the diameter of the fiber, and the use of the fiber with or without water spray cooling appear to influence the occurrence of carbonization and cracks [22, 23]. The obturation of a greater number of root canal ramifications using gutta-percha and/or sealer after treatment with Er,Cr:YSGG following mechanical instrumentation has also been demonstrated [24]. The influence of morphological changes, due to the use of the Er,Cr:YSGG laser, on the marginal sealing of the root canal obturation has not yet been investigated. The purpose of this study is therefore to evaluate the marginal sealing of root canal obturations previously treated with laser before and also after apical resection.

## 2. Material and Methods

### 2.1. Tooth Selection

75 extracted human straight single-rooted teeth with mature apices were selected for the leakage experiments (5 groups of 15 teeth) and an additional 15 teeth (5 groups of 3 teeth) for scanning electron microscopic evaluation. The distribution of the teeth among the experimental groups for the leakage assessment is shown in Table 1. All teeth were stored in 10% formalin until each experimental subgroup was completed. The preservation time before root canal treatment did not exceed 6 weeks. All teeth were radiographed from two angles before root canal treatment in order to exclude teeth with multiple root canals. Organic debris was removed by submerging the teeth in 2.5% sodium hypochlorite for 8 h. Subsequently, they were washed with tap water for 1 h, and stored in saline solution until used. The experimental protocol was approved by the ethical committee of the Ghent University Hospital (Belgium) (2008/627).

### 2.2. Root Canal Treatment

Crowns were removed 2 mm above the cementoenamel junction using a high-speed fissure bur and water spray. After gross removal of pulp tissues, a size 10 Flexofile (Dentsply Maillefer, Baillaigues, Switzerland) was introduced into the canal until it could be seen in the major apical foramen. The working length was determined by subtracting 1 mm from this length. The root canals were prepared by one operator using a crowndown/stepback technique. The coronal half of the root canals was preflared with Gates Glidden drills (Dentsply Maillefer) in a larger to smaller sequence (numbers 4-3-2). The root canals were irrigated with 2.5% sodium hypochlorite solution using a 27-gauge Endodontic Needle (Monoject, Sherwood Medical, St Louis, MO, USA). The apical half of the canal was prepared after coronal preflaring with the stepback technique up to a master file size 40 and additional files up to a size 60. The canals were dried with paper points and the patency of the apical foramen was confirmed with a size 10 Flexofile.

Five groups of 15 teeth (groups 1 to 5) were made based on the irrigation protocol and the laser irradiation approach as follows: (1) 2.5% NaOCl rinses during root canal preparation and a final rinse with 17% EDTA (Pulpdent EDTA Solution 17%, Pulpdent Corporation, Watertown, MA, USA) for 3 minutes (standard protocol), followed by the rinsing out of the 17% EDTA with 2.5% NaOCl; (2) standard protocol + Er,Cr:YSGG (Millennium Biolase Technology Inc., San Clemente, CA, USA) lasing in a dried root canal; (3) 2.5% NaOCl rinses during root canal preparation followed by Er,Cr:YSGG lasing in a dried root canal; (4) standard protocol + Er,Cr:YSGG lasing in a wet root canal; and (5) 2.5% NaOCl rinses during root canal preparation followed by Er,Cr:YSGG lasing in a wet root canal. These five groups consisted of the nonresected teeth. The same teeth were then horizontally resected up to the most apical point of the canal preparation, so that the root canal filling was exposed.

### 2.3. Laser Treatment

Root canals in the lased groups were irradiated with an Er,Cr:YSGG laser (2.796 *μ*m) with a flexible fiber [diameter 300 *μ*m − Z3 Endolase (Biolase)] at 1.5 W, 20 Hz, 75 mJ, and a 100% air pressure. The flexible fiber was inserted into the root canal one millimeter short of the working length. During irradiation, the fiber tip was moved in a spiral motion along the root canal walls. The procedure was repeated five times for 5 s with a time interval of 20 s. All apical foramina remained patent (control with a file ISO 15). The present procedure will be referred to as the conventional approach, that is, a spiral motion along the root canal wall. This study also serves as a reference for a second study where the effect of laser activated irrigation (i.e., the influence of a bubble stream) on the marginal seal of root fillings will be investigated.

### 2.4. Root Canal Filling

All root canals were dried with paper points before filling with the cold lateral condensation technique. A standard size gutta-percha cone (Dentsply Maillefer) that matched the master apical file was fitted to the working length with tug back. Root canal sealer AH 26 (Dentsply Detrey, Konstanz, Germany) was mixed according to the manufacturer's instructions and placed in the canal with the gutta-percha to the working length. The master cone was then coated again with root canal sealer and gently seated at the working length.

Lateral condensation was carried out using size 20 and 25 accessory gutta-percha cones with endodontic finger spreaders (Dentsply Maillefer) placed in the first instance to within 1 mm of the working length. The gutta-percha cones coated with sealer were laterally condensed until they could not be introduced more than 3 mm into the root canal. Following obturation, the gutta-percha was removed from the coronal cavity up to the level of the cementoenamel junction with a warm instrument (PK Thomas Waxing Instrument, N° PKT-2, Hu Friedy, Leimen, Germany) and vertically condensed with Machtou pluggers (Dentsply Maillefer).

After the root filling procedure, a small cotton pellet was sealed in the access cavity of all root-filled teeth using Ketac-Fil (3MEspe, Seefeld, Germany). The samples were then stored in Vacutainers at 80% relative humidity for 48 h at 37°C (start of the first capillary flow porometry measurements). Before storing the teeth, radiographs were taken from the buccal and mesial sides of every tooth.

### 2.5. Measurement of Capillary Flow

**Figure 1:**
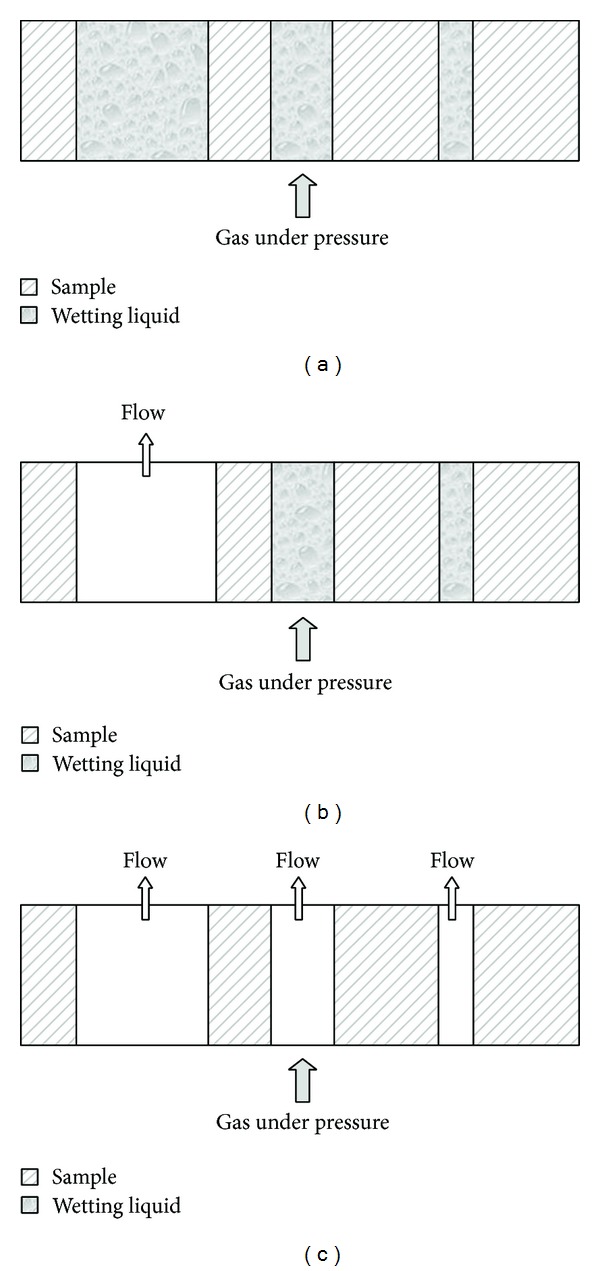
Principle of capillary flow porometry. As a result of gas pressure exerted on the sample (a), the largest existing pore is emptied first through which the flow is now measured (b). Then, in a descending order, smaller pores will be emptied until all the pores are empty (c).

Capillary flow porometry (CFP-1200-A, PMI, New York, NY, USA) provides fully automated through pores analysis including bubble point pressure, pore size distribution, and mean pore size. A wetting liquid (Galwick: 15.9 Dynes/cm, PMI) was used to fill the pores of the sample. The fully wetted teeth were attached in the sample chamber (Tubepack, Legris Connectic, France), with adhesive epoxy (Loctite 3430, Loctite, Kontich, Belgium), after which the sample chamber was sealed. Gas was then allowed to flow into the chamber behind the sample (Figure 1). When the pressure reaches a point that can overcome the capillary action of the fluid within a pore (maximum pore), the equivalent bubble point pressure has been found. After determination of the bubble point pressure, the pressure is increased and the flow is measured until all pores are empty, and the sample is considered dry. Pressure ranges from 0 to 200 PSI and the pore size range that can be measured lies between 0.035 and 500 microns. The validity of this technique in dentistry has been verified by De Bruyne et al. [23, 24]. Measurements were performed at VITO (Vlaamse Instelling voor Technologisch Onderzoek, Mol, Belgium).

After 48 hours, all teeth (groups 1 to 5) were measured after removal of the Ketac-Fil filling and cotton pellet, in order to assess the minimal, mean, and maximal through-pore diameters of each experimental tooth. Voids responsible for leakage were supposed to be present between the root canal filling and the root itself.

A second series of measurements was performed after resection of the root end up to the most apical point of the preparation length, so that the gutta-percha was exposed. In most cases, this resulted in the resection of at least 1 mm from the physiological apex and almost 2 mm from the root tip seen as the radiological apex on a radiograph. The resection was performed with a diamond wheel saw. After this procedure, all teeth (now groups 6 to 10) were subjected again to CFP. So, a comparison can be made between teeth with an apical constriction and those where the apical gutta-percha is exposed as is the case with resected teeth.

### 2.6. Statistical Analysis

Results from both methods were analyzed statistically using nonparametric tests; comparison between the leakage results according to the different additional cleaning protocols was made with Kruskal-Wallis and Mann-Whithney *U* tests. The level of significance was set at 0.05.

*Scanning Electron Microscopic Evaluation*. Limited information exists regarding the morphologic changes following Er,Cr:YSGG laser irradiation in root canals after irrigation with both NaOCl and EDTA. In order to visualize the effect of the cleaning protocol on the root canal walls, SEM analysis was performed on all experimental groups. Three additional teeth from each experimental group were analyzed by SEM [25]. Using small rotating discs, deep grooves were cut on the buccal and palatal surfaces without perforating the root canal. The roots were then split with a sharp chisel and a hammer. Care was taken to include the apical foramen in the fracture line. The samples were then dehydrated in ascending series of aqueous ethanol, critically point dried with liquid CO_2_, sputter coated with gold (JEOL JFC1200, JEOL LTD, Japan), and examined under the scanning electron microscope (JEOL JSM-5600-LV, JEOL LTD, Japan).

Representative microphotographs were taken by an independent blind investigator at 2000x magnification at 1, 3, 6, 8, and 12 mm from the apical extent of the preparation.

## 3. Results

### 3.1. Leakage Assessment by Means of Capillary Flow Porometry

Measurements were obtained for each sample at each point in time, confirming the presence of through pores regardless of which root canal wall cleaning protocol was being tested. Exact values for minimum, mean flow, and maximum pore diameters (range and median) of each sample were obtained. The results of the study are summarized in Table 2.

No statistically significant differences were found from all groups, from the groups submitted to laser treatment to those without, the groups with dried and wet root canals, and from nonresected and resected groups (*P* > 0.05).

### 3.2. Scanning Electron Microscopic Evaluation

**Figure 2:**
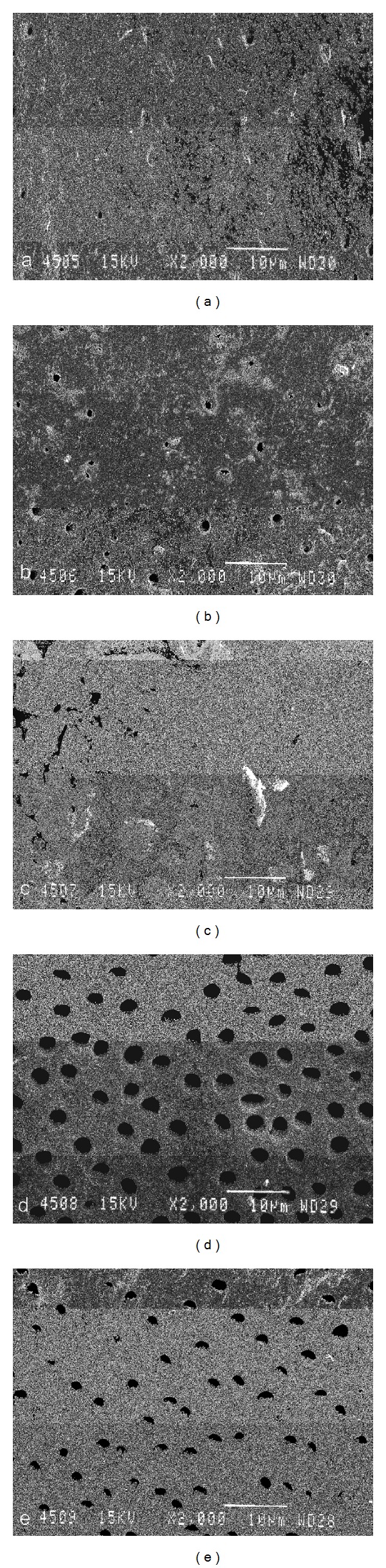
Photomicrographs of root canal wall in each group after final cleaning protocols (original magnification: 2000x; scalebar is 10 *μ*m). Images (a)–(e) show canal walls at 1, 3, 6, 8, and 12 mm from the apex. In groups 1 and 6, a typical amorphous smear layer on root canal wall, with limited opening of the dentinal tubules, was observed up to 3 mm from the apical preparation point. At 6 mm, remnants of debris and smear layer were observed. (a) is at 1 mm from the apex and (e) is at 12 mm from the apex.

**Figure 3:**
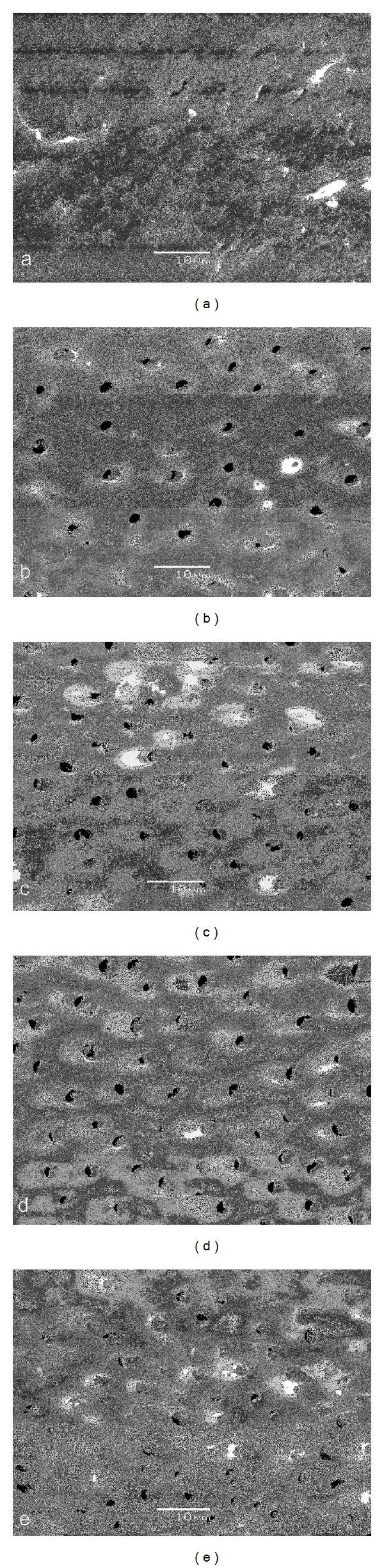
Photomicrographs of root canal wall in each group after final cleaning protocols (original magnification: 2000x; scalebar is 10 *μ*m). Images (a)–(e) show canal walls at 1, 3, 6, 8, and 12 mm from the apex. In groups 2 and 7, open tubules and removal of smear layer were clearly observed at all sites except at 1 mm. White areas of erosion around the dentinal tubule openings were seen. (a) is at 1 mm from the apex and (e) is at 12 mm from the apex.

**Figure 4:**
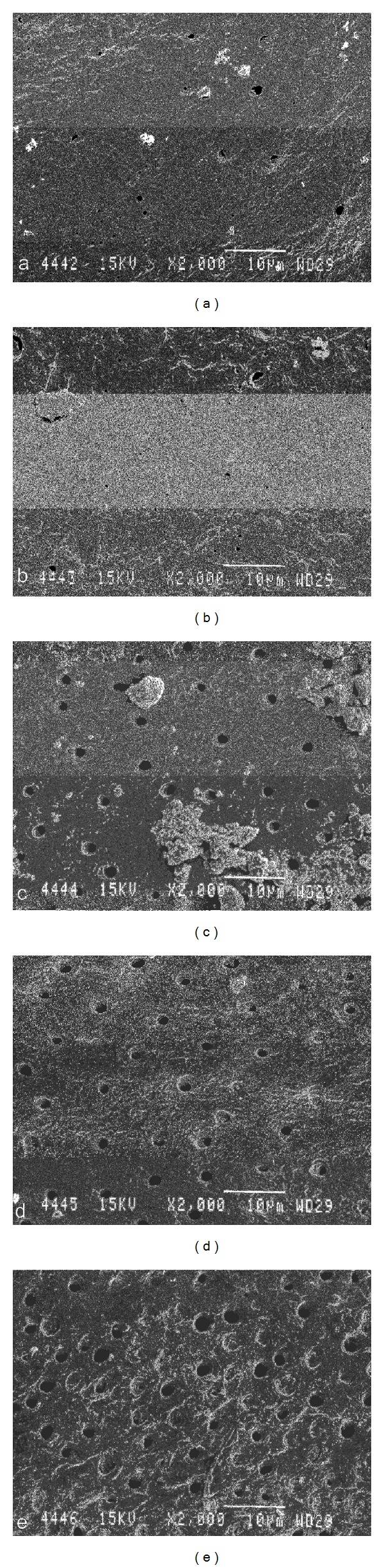
Photomicrographs of root canal wall in each group after final cleaning protocols (original magnification: 2000x; scalebar is 10 *μ*m). Images (a)–(e) show canal walls at 1, 3, 6, 8, and 12 mm from the apex. In groups 3 and 8, smear layer removal was observed at 6, 8, and 12 mm, though less extensive than in groups 2 and 7 (Figure 3) and 4 and 9 (Figure 5). (a) is at 1 mm from the apex, (e) is at 12 mm from the apex.

**Figure 5:**
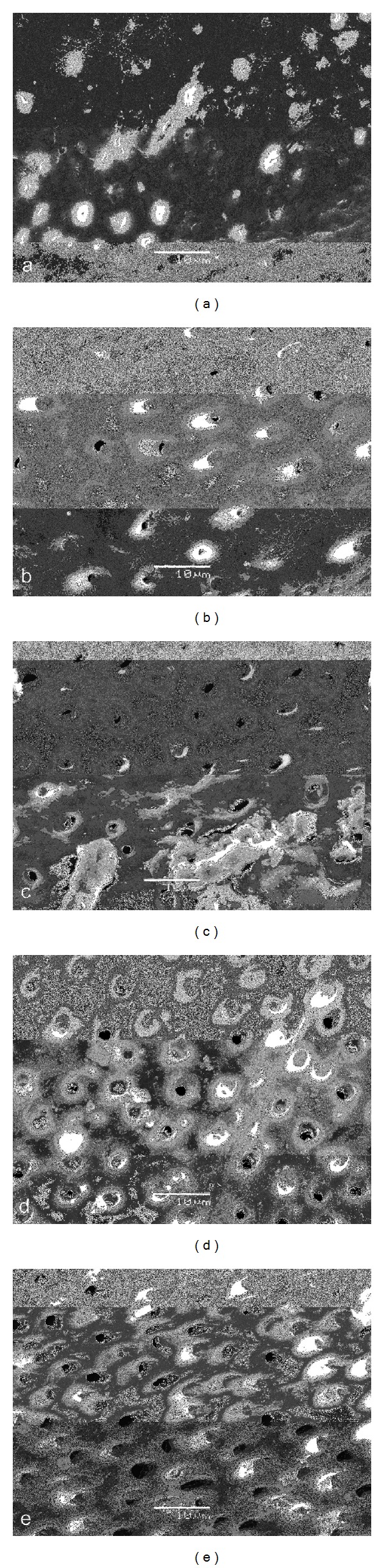
Photomicrographs of root canal wall in each group after final cleaning protocols (original magnification: 2000x; scalebar is 10 *μ*m). Images (a)–(e) show canal walls at 1, 3, 6, 8, and 12 mm from the apex. In groups 4 and 9, open tubules and removal of smear layer were clearly observed at all sites except at 1 mm. White areas of erosion around the dentinal tubule openings were seen. (a) is at 1 mm from the apex and (e) is at 12 mm from the apex.

**Figure 6:**
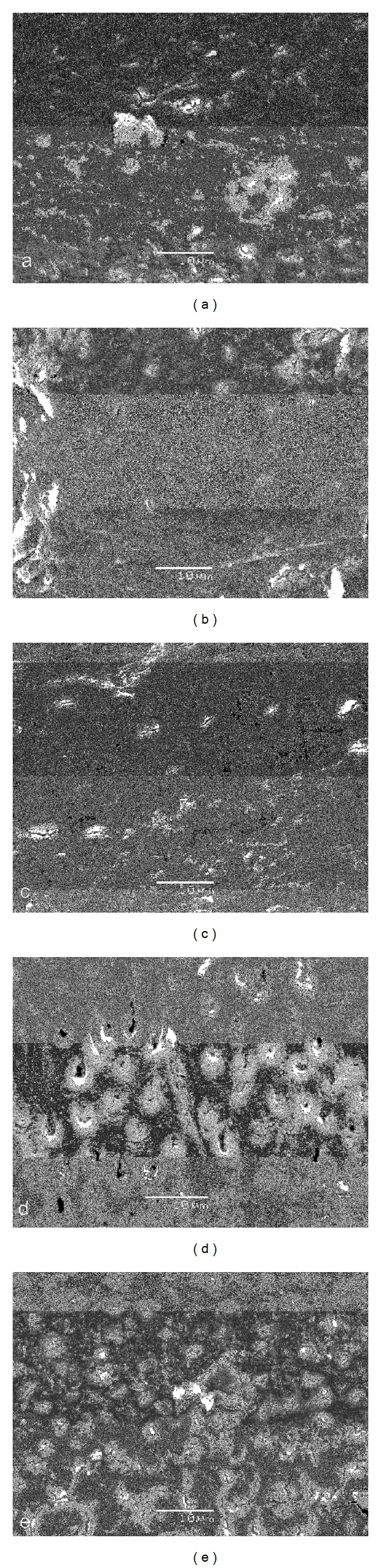
Photomicrographs of root canal wall in each group after final cleaning protocols (original magnification: 2000x; scalebar is 10 *μ*m). Images (a)–(e) show canal walls at 1, 3, 6, 8, and 12 mm from the apex. A dense and heterogeneous smear layer covering the entire dentinal wall was observed in groups 5 and 10. (a) is at 1 mm from the apex, and (e) is at 12 mm from the apex.

Figures 2 to 6 give an overview of representative images of the groups with the final cleaning protocols. In groups 1 and 6, a dense and homogeneous smear layer covering the dentinal surface was observed, and occasionally some open dentinal tubules in the apical 3 mm (Figures 2(a) and 2(b)). Remnants of debris were still observed at 6 mm, but at 8 and 12 mm, smear layer and debris free root canal walls were seen. Both groups 2 and 4 showed comparable results (Figures 3 and 5/groups 2 and 7, and 4 and 9). The removal of smear layer was seen at all sites except for the apical 1 mm. White areas of erosion around the orifices of the dentinal tubules were also observed. In groups 3 and 8, smear layer removal was observed at 6 mm and higher up along the root canal wall; no erosion around the orifices of the dentinal tubules was present (Figure 4). These findings are in contrast with the microphotographs in groups 5 and 10 where a dense and heterogeneous smear layer was present at all levels (Figures 6(a) to 6(e)).

It has also to be mentioned that no carbonization effects were found in the lased groups.

## 4. Discussion

The Er,Cr:YSGG laser is one of the more recently introduced wavelengths in endodontics with its FDA approval in 2002. Where the information on the use of Er:YAG laser in conventional mode, that is, spiral motion along the root canal wall, is more elaborate, this is not the case for the Er,Cr:YSGG laser [1, 2]. Information on its influence on root canal filling quality is limited [22] and lacking on its influence on the marginal sealing of root canal obturations.

In this study, capillary flow porometry (CFP) was used to assess leakage. This method was introduced in endodontics by De Bruyne et al. [23, 24]. CFP was chosen as the evaluation method because of its nondestructive nature and the highly reproducible and accurate data it generates [26, 27]. As such, the method can overcome the problem of limited reproducibility and comparability of conventional methods for evaluating leakage.

Chemomechanical preparation of the root canal creates smear layer and debris which consist of dentin chips and remnants of organic material [28, 29]. This layer acts as a physical barrier, occludes dentinal tubules, harbors microorganisms, and does not prevent bacterial migration into tubules [30–33]. The alternate use of NaOCl and EDTA irrigants results in smear layer removal and a dentin surface with open tubules [34]. As a consequence, a better interaction of irrigants and intracanal medication with the dentinal root canal walls, remaining microorganisms, and the remains of the biofilm becomes possible; open tubules result in a deeper penetration of root canal sealer and more obturated lateral canals [35–38]. The removal of smear layer in the apical region, however, remains unpredictable [39, 40]. The latter was confirmed during the SEM examination in all groups of the present study (Figures 2–6).

The Er,Cr:YSGG is a laser system very similar to that of Er:YAG laser and shows similar performance with the Er:YAG on mineralized tissues [41, 42]. While the topographic and thermographic effects of Er,Cr:YSGG laser and its suitability for etching enamel surfaces have been studied in detail [43], the reports since 2002 also focus on the application in root canal treatment [17, 18, 22, 44–48]. Typical ultramorphological changes with the Er,Cr:YSGG that have been reported are partial or total removal of dentinal smear, as well as regions of exposed tufted collagen, masking tubule orifices; the presence of sites showing thermal injury, including carbonization and partial melting [17, 18, 44, 45]. The use of a water mist during ablation with the Er,Cr:YSGG laser was emphasized in order to avoid cracks and carbonization and achieve successful removal of the smear layer and debris [20]. No areas with signs of carbonization were discovered on the dentinal root canal walls, demonstrating that the temperature developed during lasing according to the instructions of the manufacturer was not of this magnitude and that damage of the periodontal ligament might occur.

In this study, however, a comparison was made between morphological changes after the use of the Er,Cr:YSGG laser in a dried root canal or in a wet canal, both with a 100% air pressure. This option was investigated based on the findings of Stabholz et al. demonstrating that erbium lasing in canals filled with 17% EDTA resulted in clean surfaces, free of smear layer and debris [49]. It is clear from Figures 3 and 5 that the presence of root canal walls impregnated with EDTA and then irradiated with the Er,Cr:YSGG laser may result in a higher cleaning efficacy (groups 2 and 7 and groups 4 and 9). Lasing into an EDTA liquid while moving the fiber along the root canal wall in a spiral motion is less efficient than in a dried canal. It is clear that the presence of the liquid interferes with the interaction of the fiber with the root canal wall dentin in the set-up of this study. The laser was also used at a far lower pulse energy (75 mJ) as compared to Stabholz et al. (500 mJ) [49]. This also accounts for lasing into the NaOCl solution where the effect of the laser apparently got lost, even when the laser was in contact with the root canal wall. In none of the lased samples signs of carbonization or melting were detected. Within the confines of this study, the investigators have collected SEM pictures in order to document the influence of laser irradiation and irrigants on root canal wall cleanliness, but are aware that the ideal experimental model to assess smear layer removal is not currently available [50]. In this respect, it was clearly emphasized by De Deus et al. that there are still unanswered questions on this issue and that the main responsible factor is the qualitative and nonreproducible character of most *in vitro* smear layer removal studies [50]. Therefore, care has to be taken with the interpretation of the data. Nevertheless, the present findings coincide with the findings of other studies aiming to remove the smear layer [17–21].

The limitations in the conventional (spiral motion of the fiber) cleaning protocol with the Er,Cr:YSGG are also due to the unidirectional emission of the laser light. In Figure 6(c) (groups 5 and 10), a track of the fiber along the root canal wall is observed demonstrating this limitation. More is to be expected from conical laser fiber tips allowing lateral emission of the laser light and/or cavitation as working mechanism of erbium lasers for the removal of smear layer and debris [1, 2].

Comparing the root canal wall cleanliness as a result of EDTA-rinses or Er,Cr:YSGG lasing (both as means for smear layer removal), it was observed that lasing was more efficient with smear layer removal up to the apical 3 mm. Clean root canal walls at 1 mm from the apex were not observed in any of the experimental groups. In the groups where lasing was performed on root canal walls impregnated with EDTA, erosion around the dentinal tubule openings was observed, demonstrating that there was a more pronounced interaction with the root canal wall dentin.

Although there were clear differences in cleaning efficacy between the 5 experimental protocols in this study, no statistically significant influence on the seal of the root canal fillings was demonstrated. In order to be sure that the apical constriction, which acts as a physical barrier, did not influence the measurements of the capillary flow porometry, the root tips were resected up to the most apical preparation point and thus exposing the apical portion of the root filling. The resection of the root tip had no statistically significant influence on the data obtained when measuring the seal of the root fillings with CFP. In this respect, it also needs to be mentioned that none of the apical constrictions demonstrated opening of the constriction area due to exposure to the Er,Cr:YSGG laser (in this study, the fiber was activated at 1 mm from the most apical preparation point before withdrawal along the root canal wall).

A three-dimensional tightly sealing root canal obturation without voids is an important parameter for a long-lasting endodontic success [51]. In a microcomputed tomography study, it has been found that laterally condensed gutta-percha restorations may contain 1.02% gaps or voids [52]. With CFP, it is possible to determine the size of the pore diameters. No statistically significant differences were measured between the mean flow pore diameter and the maximum pore diameter. The maximum pore diameter is the most important determinant for the quality of the apical seal. Knowing that the average length of bacteria varies between 0.2 and 1.5 *μ*m, and that these sizes for toxins are even smaller [53], it is clear from Table 2 that bacteria can pass along the root filling in the different experimental groups. Apparently, a better cleaning protocol as provided with the Er,Cr:YSGG after a final EDTA-rinse does not necessarily imply a better seal of the root canal filling even when more dentinal tubules are open for sealer penetration.

## 5. Conclusion

Recognizing the inherent limitations of an *in vitro* experiment, the use of a final 17% EDTA-rinse or the use of the Er,Cr:YSGG laser (spiral motion along the root canal wall) was less efficient in removing dentin debris and smear layer than when the laser was additionally used after a final 17% EDTA-rinse. Under the conditions of the present study, the effect of the Er,Cr:YSGG was more pronounced in dried root canals, then when the fiber was used in an EDTA or a NaOCl solution.

Cleaner root canal walls apparently did not result in a better seal of the laterally condensed gutta-percha root filling when capillary flow porometry was used for the determination of through pores.

## Figures and Tables

**Table 1:** Distribution of the teeth among the experimental subgroups (1–10).

	Nonresected roots	Resected roots
Control group	1—NaOCl + EDTA	6—NaOCl + EDTA
Dry lasing protocol	2—NaOCl + EDTA + Er,Cr:YSGG	7—NaOCl + EDTA + Er,Cr:YSGG
	3—NaOCl + Er,Cr:YSGG	8—NaOCl + Er,Cr:YSGG
Wet lasing protocol	4—NaOCl + EDTA + Er,Cr:YSGG	9—NaOCl + EDTA + Er,Cr:YSGG
	5—NaOCl + Er,Cr:YSGG	10—NaOCl + Er,Cr:YSGG

**Table 2:** Range and median of minimum, mean flow, and maximum pore diameters by root end filling material at 48 h (groups 1 to 5) and then immediately after root resection (groups 6 to 10).

Group	Minimum pore diameter (*μ*m)		Mean flow pore diameter (*μ*m)		Maximum pore diameter (*μ*m)	
	Range	Median	Range	Median	Range	Median
1	0.069–0.183	0.0870	0.078–0.277	0.1080	0.152–0.483	0.3020
2	0.069–0.172	0.0820	0.075–0.237	0.1400	0.100–0.558	0.2510
3	0.069–0.238	0.0710	0.073–0.332	0.1220	0.126–0.414	0.2910
4	0.069–0.410	0.0980	0.079–0.449	0.1660	0.143–0.881	0.3340
5	0.069–0.112	0.0760	0.078–0.194	0.091	0.094–0.550	0.1830
6	0.069–0.209	0.0840	0.090–0.332	0.1180	0.162–0.976	0.3080
7	0.069–0.244	0.0870	0.074–0.266	0.1420	0.144–0.452	0.2840
8	0.069–0.171	0.0710	0.075–0.247	0.0850	0.170–0.486	0.2560
9	0.069–0.212	0.1020	0.074–0.520	0.1170	0.119–1.289	0.2640
10	0.069–0.148	0.0720	0.077–0.256	0.1120	0.158–0.641	0.2670

Groups: 1: irrigation with NaOCl 2.5% and EDTA 17% or standard protocol (SP), 2: SP + Er,Cr:YSGG lasing (dried root canal-RC), 3: NaOCl 2.5% + Er,Cr:YSGG lasing (dried RC), 4: SP + Er,Cr:YSGG lasing in EDTA (wet RC), and 5: NaOCl 2.5% + Er,Cr:YSGG lasing in NaOCl (wet RC). Groups 6 to 10 consist of the filled teeth of groups 1 to 5 with resected apices up to the most apical point of the preparation length and exposing the root canal filling.
